# Voice-Based Structured Nursing Documentation Using Automatic Speech Recognition and Large Language Models: Development and Evaluation Study

**DOI:** 10.2196/88567

**Published:** 2026-06-05

**Authors:** Meng-Han Su, Wei-Chun Wang, Yi-Min Hsu, Shih-Yen Hou, Su-Jung Chuang, Shih-Sheng Chang

**Affiliations:** 1 Artificial Intelligence and Robotics Innovation Center, China Medical University Hospital, China Medical University, No. 2, Yude Rd, North Dist, Taichung, 404327, Taiwan, 886 0422052121 ext 12584; 2 China Medical University Hospital, Taichung, Taiwan

**Keywords:** automatic speech recognition, code-switching, large language model, nursing, documentation, nursing records

## Abstract

**Background:**

For clinical nurses, manually entering information into hospital information systems (HISs) remains time-consuming and prone to omissions. Although speech recognition can reduce the need for manual entry, its use in clinical settings has historically been limited by code-switching, medical terminology, and noisy ward environments. Recent advances in customized automatic speech recognition (ASR) and large language models (LLMs) now make speech-based, structured documentation aligned with nursing frameworks such as DART (data, action, response, and teaching) increasingly feasible.

**Objective:**

This study developed and evaluated an integrated ASR and LLM system that transforms spoken nursing input into structured DART notes and evaluated its accuracy, usability, and clinical feasibility within HIS workflows.

**Methods:**

A code-switching nursing speech corpus from emergency and ward settings was used to fine-tune the Whisper large-v2 model with parameter-efficient adaptation. The LLM generated schema-constrained DART records from ASR transcripts, which were verified by nurses before being uploaded to the corresponding HIS fields. Evaluation included mixed error rate for ASR accuracy, *F*_1_-scores, and agreement statistics for DART classification, hallucination assessments based on factual correctness, and analysis of nurse feedback on system use.

**Results:**

The fine-tuned ASR model reduced the mixed error rate from 44.79% to 6.67%. DART generation achieved a macroaveraged *F*_1_-score of 0.82 (95% CI 0.80‐0.84) and met the noninferiority margin relative to human transcripts (δ=−0.04). The hallucination rate was 2.51%. During deployment, the monthly volume of valid nursing notes generated through voluntary use of the ASR system increased from 32,724 to 65,417, where each note represented a single documentation entry generated per patient care episode. Among 120 participating nurses, 91 (75.8%) reported reduced workload and improved completeness.

**Conclusions:**

The integrated ASR and LLM system was feasible and showed strong performance, with good acceptance among clinical nurses. It reduced the manual documentation burden, improved record completeness, and demonstrated the value of an ASR- and LLM-supported workflow for nursing documentation.

## Introduction

Taiwan has faced a sustained shortage of clinical nurses, driven largely by low retention among licensed professionals rather than limited training capacity. The national nursing practice rate—representing the percentage of licensed nurses actively working in the profession—remains at only 59.1% [1]. This low retention of qualified staff exacerbates the clinical shortage, and excessive workloads and long shifts are strongly associated with burnout and turnover intention [2-4]. Documentation occupies a substantial portion of clinical workflow time, and existing digital systems have not fully reduced the need for manual input. As hospitals have expanded the use of hospital information systems (HISs), additional documentation requirements have emerged, prompting efforts to standardize formats and improve system integration [5]. Structured and interoperable records support continuity of care, and structured handoff protocols improve communication quality [6,7]. Moreover, higher perceived nursing information system quality is linked to greater use, satisfaction, and retention, while electronic record use embedded in routine workflows reduces documentation workload and intention to leave [8,9]. These findings highlight the need for documentation tools that are structured, interoperable, and well-integrated into daily clinical practice.

Many hospitals have introduced structured documentation frameworks to improve consistency and communication. Among these, the Focus Charting method has been particularly influential in Taiwan. Originating in the late 1980s [10] and adopted locally in the early 1990s [11], it organizes each nursing note around a defined patient focus and follows the DART (data, action, response, and teaching) pattern to support concise and standardized recording of observations, interventions, patient responses, and teaching activities. Implementation studies have reported clearer note organization, improved execution of care plans, and enhanced interdisciplinary collaboration in intensive care settings [12]. However, structured formats alone do not resolve the documentation burden. Even with electronic templates, nurses must still enter most information manually, and flowsheets—standardized tabular records used for tracking routine, time-sequenced clinical parameters such as vital signs—may occupy a substantial portion of each shift [13]. These limitations highlight the need for intelligent support that preserves the clarity of DART records while reducing repetitive manual effort.

Automatic speech recognition (ASR) has been explored as a way to reduce manual documentation effort, with multisite evaluations reporting higher efficiency and satisfaction [14,15]. In Taiwan, a longitudinal study involving 21 nurses (using a corpus of 30,112 words) found that mean accuracy improved from 87.06% to 95.07% across 4 evaluation sessions as users developed more stable speaking speeds and volumes [16]. Furthermore, an ASR system designed for code-switching—the practice of alternating between languages, such as Mandarin and English, within a single utterance—achieved a word error rate (a standard metric representing the percentage of transcription errors) of 12.3% in intensive care settings [17].

Recent reviews suggest that clinical speech technologies are rapidly evolving toward large language model (LLM)–driven ambient clinical intelligence, with mature commercial solutions (such as Heidi, Tortus, and Rebrief.ai [Rebrief Inc]) being successfully deployed in various settings to provide summarization, repurposing, and assistive autonomy [18]. However, although these ambient scribes excel in standard clinical encounters, their direct application to Taiwanese nursing workflows presents significant challenges. First, commercial systems are primarily optimized for monolingual or standard bilingual speech and often struggle with the dense, specialized Mandarin-English code-switching and local abbreviations prevalent in Taiwanese hospital wards. Second, existing tools typically generate generalized clinical summaries (eg, subjective, objective, assessment, and plan notes), lacking the fine-grained, schema-constrained precision required for specialized nursing frameworks such as DART and its direct interoperability with local HISs. Therefore, a bespoke ASR and LLM pipeline is necessary to address these specific linguistic and structural demands. To bridge this gap, this study developed and evaluated an integrated voice-to-report system that combines a domain-adapted ASR model with an LLM to generate structured DART nursing documentation aligned with HIS fields. The system was assessed using transcription accuracy, DART classification performance, and noninferiority testing, and its feasibility and workflow impact were examined during real-world deployment.

## Methods

### Study Design and System Overview

**Figure 1.**
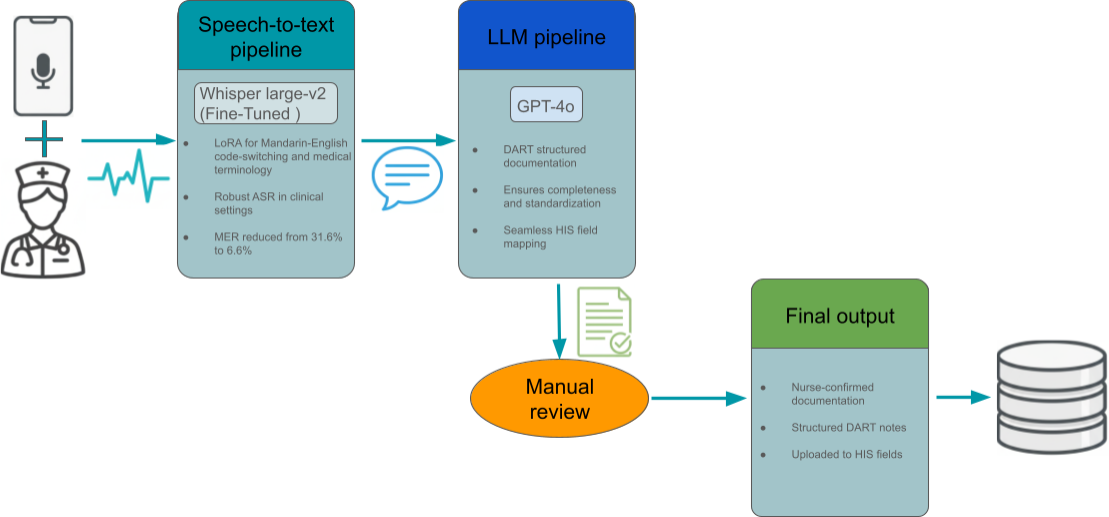
Workflow of the voice-to-DART (data, action, response, and teaching) documentation system. ASR: automatic speech recognition; HIS: hospital information system; LLM: large language model; LoRA: low-rank adaptation; MER: mixed error rate.

This development and evaluation study implemented a mobile-based nursing documentation system designed to convert voice input into structured clinical records (Figure 1). The system was deployed at China Medical University Hospital (CMUH) to evaluate its feasibility, transcription accuracy, and impact on documentation workflow. During routine care, nurses recorded observations and interventions using a mobile device. Audio input was processed by a domain-adapted Whisper ASR model trained on a Mandarin-English code-switching corpus and adapted to the medical terminology common in nursing communication. Transcribed text was subsequently processed by GPT-4o (OpenAI) using a schema-constrained prompt to generate standardized DART notes primarily in Mandarin while preserving English medical terminology. Nurses reviewed the generated content and triggered its upload to predefined HIS fields.

The system was developed in-house by the Artificial Intelligence and Robotics Innovation Center at CMUH. The codebase and intellectual property are proprietary. Due to strict patient privacy regulations and direct HIS integration, the software and raw datasets are not publicly available. However, to ensure methodological reproducibility, all model hyperparameters, evaluation frameworks, and complete schema-constrained prompt templates are fully detailed in this paper and Multimedia Appendix 1.

### Dataset and Ground-Truth Preparation

The ASR model was trained and evaluated on nursing speech data from 3 complementary sources. The primary corpus, CMaiSpeech, comprised spontaneous Mandarin-English code-switched recordings from 525 nurses across multiple clinical units, characterized by frequent English medical terms and abbreviations. Table 1 summarizes the counts and durations of CMaiSpeech by category. Two synthetic corpora (approximately 9 hours) were generated using neural text-to-speech models from 1838 drug names and 1566 clinical terms to enrich underrepresented medical vocabulary. A real-world clinical dataset (totaling approximately 6 hours, 1608 utterances) captured authentic ward conversations, including vital-sign reporting and handovers. All recordings were normalized and resampled to 16-kHz pulse code modulation to ensure acoustic consistency. These datasets collectively served as the domain-adapted speech material used for training and evaluating the Whisper-based ASR model in nursing contexts.

In addition, a validation dataset of 327 nurse-authored documentation samples was constructed. Each sample included an audio recording collected during routine care, a manually transcribed reference serving as the ASR gold standard, and structured DART annotations verified by clinical nurses. This dataset was used to evaluate ASR transcription accuracy, LLM-based DART structuring, and agreement between fine-tuned and zero-shot conditions under a consistent reference standard.

**Table 1.** Counts and audio durations by category in the CMaiSpeech dataset.

Category	Item count (N=7055), n (%)	Audio length (seconds)^a^
Diastolic BP^b^	20 (0.28)	36.57
Glasgow Coma Scale eye response component	20 (0.28)	99.61
Pain index	20 (0.28)	46.05
Pulse	20 (0.28)	34.27
Systolic BP	20 (0.28)	39.34
Body temperature	20 (0.28)	39.10
Vital signs^c^	1035 (14.67)	12,350.10
Nursing notes^c^	5900 (83.65)	75,149.10

a Total duration of all audio samples within each category.

b BP: blood pressure.

c Categories representing full-length clinical recordings captured during routine nursing documentation activities.

### ASR Model Adaptation and Evaluation

Whisper is a multilingual Transformer-based ASR model pretrained on 680,000 hours of diverse speech data [19]. Although its large-scale pretraining supports robust zero-shot generalization, accuracy declines in nursing-specific communication, where frequent Mandarin-English code-switching, dense medical terminology, and numerous drug abbreviations remain underrepresented. These discrepancies limit its direct applicability to structured nursing documentation.

Prior studies have demonstrated that domain-targeted fine-tuning markedly improves Whisper’s performance in medical and mixed-language contexts. Previous studies reported gains in ASR and named entity recognition on Mandarin speech [20], and further improvements were achieved using domain-adapted fine-tuning and prompting on Mandarin-English medical data [21]. Building on these findings, Whisper (large-v2; OpenAI) was adapted to the nursing domain through low-rank adaptation–based parameter-efficient fine-tuning, which introduced trainable low-rank matrices into frozen weights to reduce computational cost while preserving performance [22]. The model was trained on a domain-specific nursing speech corpus and evaluated using mixed error rate (MER) to assess bilingual transcription accuracy relative to the baseline model.

Prior to MER calculation, a rigorous text normalization pipeline was applied to both reference and hypothesis transcripts to ensure consistent scoring. All full-width alphanumeric characters were converted to half-width equivalents, and punctuation marks were removed. Original casing for English text and medical abbreviations (eg, “SpO_2_” and “BP”) was strictly preserved to evaluate the model’s ability to output correctly capitalized clinical terms. Crucially, numeric values, including decimals, were evaluated as independent, contiguous tokens rather than as split characters to accurately reflect their semantic weight in clinical parameters such as vital signs.

### LLM Integration and Evaluation

The LLM was integrated to convert ASR transcripts into structured nursing documentation following the DART schema. GPT-4o (accessed via Azure OpenAI, application programming interface version 2024-12-01-preview), a multimodal variant of the GPT-4o family optimized for instruction following and multilingual text generation, was used for schema-based text structuring [23]. The model hyperparameters were consistently set to a temperature of 0.7, a top_p of 0.95, and a maximum of 16,000 tokens. A schema-constrained prompting strategy was applied to ensure consistent field separation and syntactic completeness, informed by prior evidence that structured prompting improves validity in clinical text generation [24]. The generation process used a dual-prompt structure: a system prompt defining the explicit task instructions and schema constraints, and a user prompt containing the ASR transcript. Two prompting configurations (a minimal version and a schema-constrained version) were compared during preliminary testing, and the schema-constrained configuration was selected for deployment. The full system prompt template is provided in Multimedia Appendix 1.

Model outputs were postprocessed to verify structural integrity and mapped to predefined HIS fields. LLM performance was evaluated on the 327-case validation dataset using field-level and macroaveraged *F*_1_-score values across DART categories. The 95% CIs were estimated using 1000 paired bootstrap resamples at the case level. Noninferiority was tested using a predefined margin of δ=0.05, with noninferiority established when the lower bound of the ∆*F*_1_-score CI exceeded −0.05. This 5% margin aligns with recent clinical evaluations of LLM-generated medical documentation, where a <5% variance is considered clinically acceptable for draft generation [25], and serves as a standard threshold in medical artificial intelligence performance evaluations [26]. From a clinical safety perspective, because the system captures data directly at the bedside via mobile devices, it inherently mitigates the high, undocumented risk of human memory decay and omission associated with traditional delayed documentation at the nursing station. Furthermore, because the system strictly requires human-in-the-loop verification prior to HIS submission, this minor structural variance during the drafting stage was deemed an acceptable trade-off for reduced documentation burden, while human oversight securely mitigates the risk of high-severity clinical errors.

### Agreement Method

Three input conditions were compared while holding the structuring LLM constant: (1) human transcripts (reference), (2) Whisper large-v2 (zero-shot), and (3) Whisper large-v2 (fine-tuned). The primary end point was the *F*_1_-score for DART slot classification against adjudicated references. A predefined noninferiority margin of δ=0.05 for Δ*F*_1_-score was applied, and 95% CIs were estimated using paired bootstrap resampling. Noninferiority was established when the lower bound of the Δ*F*_1_-score CI exceeded −0.05.

Field-level consistency was assessed because the “data,” “action,” “response,” and “teaching” components of the DART framework represent distinct documentation intents. Labels were binarized (present-or-absent) after whitespace trimming, and both percent agreement and Cohen κ were calculated to adjust for chance agreement under skewed prevalence distributions [27].

It should be noted that the binarized *F*_1_-score and agreement metrics were specifically chosen to evaluate the system’s capability in initial structural triaging, ensuring that the LLM correctly maps spoken intents to the corresponding DART fields. Because the system operates within a strict human-in-the-loop workflow in which nurses must review and adjust the drafted text prior to HIS submission, achieving high structural accuracy significantly reduces manual documentation burden, even if minor within-field reallocations are occasionally required. To address content accuracy and penalize clinically incorrect information within these fields, the FactualCorrectness metric was used separately during the hallucination assessment.

### Hallucination Assessment

Hallucination was defined as any generated content that lacked support from the corresponding reference transcript for the same case. Evaluation followed the FactualCorrectness metric from the Retrieval-Augmented Generation Assessment (RAGAS) framework (precision mode), which decomposes model output into atomic claims and verifies each claim against the reference transcript [28,29]. A fixed Azure OpenAI model (GPT-4o, application programming interface version 2024-12-01-preview; temperature=0.7, top_p=0.95) was used as the evaluator via a LangChain wrapper to ensure consistent claim-level scoring. For each case, the 4 DART fields were concatenated into a single response, and both generated and reference texts were normalized prior to evaluation.

Mean factual-correctness precision and its complement (hallucination rate) were reported, along with the proportion of notes exhibiting hallucination under strict (<1.00) and relaxed (<0.95) thresholds. All computations were performed in Python (version 3.12; Python Software Foundation) using the RAGAS library [30].

### System Use and User Feedback

To evaluate real-world feasibility and system adoption, use metrics were monitored during the initial deployment period at CMUH. Monthly use was quantified by extracting the total number of system-generated records from the application logs between March 2025 and August 2025.

Additionally, user feedback was collected to assess system acceptability and its impact on clinical workflow. Nursing staff from diverse clinical units, including wards and intensive care departments, voluntarily provided evaluations after integrating the system into their routine practice. The evaluation mechanism captured both quantitative satisfaction ratings (categorized as favorable or dissatisfied) and qualitative comments regarding user experience. Descriptive statistics were used to summarize the quantitative use and satisfaction data, while qualitative feedback underwent thematic analysis to identify common themes related to documentation efficiency, manual workload, and system accuracy.

### Ethical Considerations

This study was approved by the Institutional Review Board of CMUH (CMUH110-REC2-181 and CMUH110-REC2-187). Since this was a retrospective study utilizing deidentified data, the requirement for informed consent was waived by the institutional review board. Patient privacy and data confidentiality were strictly maintained throughout the study, and no compensation was involved.

## Results

### Evaluation Dataset Characteristics

The evaluation dataset comprised 327 annotated nursing documentation samples, as described in the Methods section. Across transcripts, the corpus contained 12,136 Chinese characters, 1361 English words, and 1130 numeric tokens, equivalent to approximately 11.2 English words per 100 Chinese characters (Table 2, panel A). A total of 540 DART annotations were identified: 257 (47.6%) for “data,” 123 (22.8%) for “action,” 123 (22.8%) for “response,” and 37 (6.9%) for “teaching” (Table 2, panel B). The lower frequency of “teaching” entries reflects routine documentation patterns and was accounted for when estimating field-specific CIs. This dataset served as the common benchmark for evaluating both ASR and LLM outputs.

**Table 2.** Token composition and DART (data, action, response, and teaching) annotation distribution in the evaluation dataset.

Panels and categories		Distribution, n (%)		Count per sample^b^, mean (SD)
Panel A: token^a^ composition (n=14,627)				
Chinese characters		12,136 (83.0)		37.11 (24.31)
English words		1361 (9.3)		4.16 (5.56)
Numeric tokens		1130 (7.7)		3.46 (4.97)
Panel B: DART annotation distribution (n=540)				
Data		257 (47.6)		0.79 (0.41)
Action		123 (22.8)		0.38 (0.49)
Response		123 (22.8)		0.38 (0.49)
Teaching		37 (6.8)		0.11 (0.32)

a Token counts were computed from manual transcripts after text normalization; Chinese tokens represent individual characters, English tokens represent space-delimited words, and numeric tokens represent digit sequences.

b Values were calculated by dividing counts by the total number of samples (n=327).

### ASR Model Performance

Whisper large-v2 was evaluated using MER, computed at the character level for Chinese and at the word level for English. In the zero-shot condition, MER was 44.79%. After low-rank adaptation–based fine-tuning on the nursing corpus, MER decreased to 6.67%, corresponding to an 85.11% relative error reduction (Table 3).

**Table 3.** Performance of Whisper large-v2 on the evaluation dataset.

Models	Mixed error rate (%)	Relative error reduction (%)
Zero-shot model^a^	44.79	—^b^
Fine-tuned model^c^	6.67	85.11

a Zero-shot: Whisper-Large-v2 inference without domain adaptation.

b Not applicable.

c Fine-tuned: Whisper-Large-v2 adapted using low-rank adaptation LoRA on the nursing speech corpus.

### DART Classification Performance

DART classification performance was evaluated under 3 input conditions: human-transcribed references, fine-tuned ASR, and zero-shot ASR (Table 4). A schema-constrained prompting strategy was used for all analyses after preliminary comparison showed higher slot-level completeness and *F*_1_-score performance than a minimal prompt.

**Table 4.** Per-field DART (data, action, response, and teaching) *F*_1_-score performance with 95% CIs under 3 input conditions.

Fields	Zero-shot^a^, *F*_1_-score (95% CI)	Fine-tuned^b^, *F*_1_-score (95% CI)	Human transcript^c^, *F*_1_-score (95% CI)
Data	0.89 (0.85‐0.92)	0.90 (0.87‐0.93)	0.91 (0.89‐0.94)
Action	0.59 (0.53‐0.66)	0.77 (0.73‐0.81)	0.83 (0.79‐0.86)
Response	0.72 (0.67‐0.77)	0.82 (0.79‐0.85)	0.85 (0.82‐0.88)
Teaching	0.62 (0.56‐0.68)	0.78 (0.73‐0.82)	0.83 (0.79‐0.86)
Macroaverage^d^	0.70 (0.68‐0.72)	0.82 (0.80‐0.84)	0.85 (0.83‐0.87)

a Zero-shot inference without domain adaptation.

b Fine-tuned using low-rank adaptation on the nursing speech corpus.

c Manually transcribed references used as the gold standard for automatic speech recognition evaluation.

d Unweighted mean *F*_1_-score across data, action, response, and teaching fields.

With this configuration, overall *F*_1_-score values were 0.85 for human transcripts, 0.82 for fine-tuned ASR, and 0.70 for zero-shot ASR. The difference between fine-tuned ASR and human input (Δ*F*_1_-score=−0.03) fell within the predefined noninferiority margin (δ=0.05). Field-level Δ*F*_1_-score values ranged from −0.01 (for “data”) to −0.06 (for “action”). Zero-shot ASR showed uniformly lower performance across all fields.

**Figure 2.**
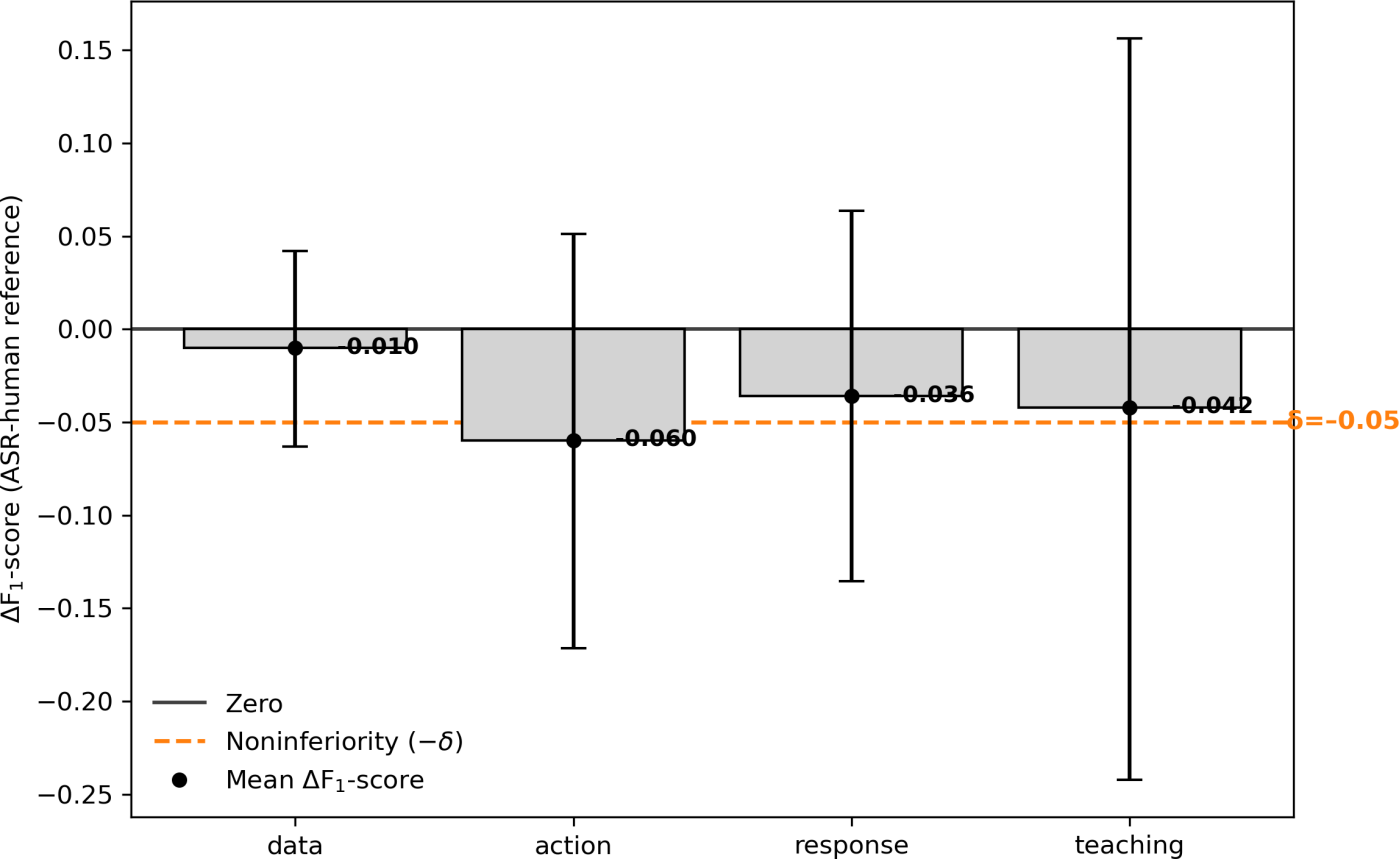
Field-level Δ*F*_1_-score values (automatic speech recognition [ASR]–human reference) with 95% CIs and the noninferiority boundary (δ=−0.05).

Figure 2 presents field-level Δ*F*_1_-score estimates with 95% CIs. All field means remained within the noninferiority boundary, supporting noninferiority at the macro level.

### Agreement Evaluation

Concordance was assessed across the 3 input conditions while holding the LLM constant. Agreement between classifications derived from the fine-tuned ASR and human transcripts ranged from 88.38% to 96.94%, with Cohen κ ranging from 0.66 to 0.86 across the “data,” “action,” “response,” and “teaching” fields (Table 5), corresponding to substantial to almost perfect agreement [28]. For the “data” field, raw agreement was 95.41% with κ=0.66, a pattern consistent with imbalanced class prevalence. Overall, slot-level results were consistent with the macrolevel noninferiority findings.

**Table 5.** Agreement and Cohen kappa for DART (data, action, response, and teaching) fields comparing large language model outputs generated from fine-tuned automatic speech recognition with outputs from human transcripts.

Fields	Agreement^a^ (%)	Cohen κ^b^
Data	95.41	0.66
Action	88.38	0.75
Response	89.91	0.79
Teaching	96.94	0.86

a Percent agreement between large language model outputs generated from fine-tuned automatic speech recognition transcripts and human transcript inputs.

b Chance-corrected agreement coefficient computed using the standard Cohen kappa formulation.

### Hallucination Evaluation

Hallucination rates were evaluated using the FactualCorrectness metric from the RAGAS framework in precision mode (Table 6). With human transcripts, the mean hallucination rate was 2.35% (SD 9.93%; 95% CI 1.27–3.43), and hallucinations were detected in 28 (8.56%) samples (95% CI 5.99–12.10). Fine-tuned ASR yielded a mean hallucination rate of 2.51% (SD 10.86%; 95% CI 1.33–3.70), with hallucinations detected in 26 (7.95%) samples (95% CI 5.48–11.40). Zero-shot ASR showed higher hallucination rates, with a mean rate of 8.98% (SD 23.99%; 95% CI 6.36–11.60) and hallucinations detected in 65 (20%) samples (95% CI 16.01–24.69).

**Table 6.** Hallucination rates of large language model outputs across different input sources.

Input sources	Hallucination rate^a^ (%), mean (SD)	Samples with hallucination^b^, n (%)
Human transcript	2.35 (9.93)	28 (8.56)
Fine-tuned ASR^c^	2.51 (10.86)	26 (7.95)
Zero-shot ASR	8.98 (23.99)	65 (20.00)

a Computed using the FactualCorrectness metric (precision mode), representing the proportion of hallucinated tokens relative to all generated tokens.

b Percentage of evaluation samples containing at least 1 hallucination (n=327).

c ASR: automatic speech recognition.

### System Use and User Feedback

**Figure 3.**
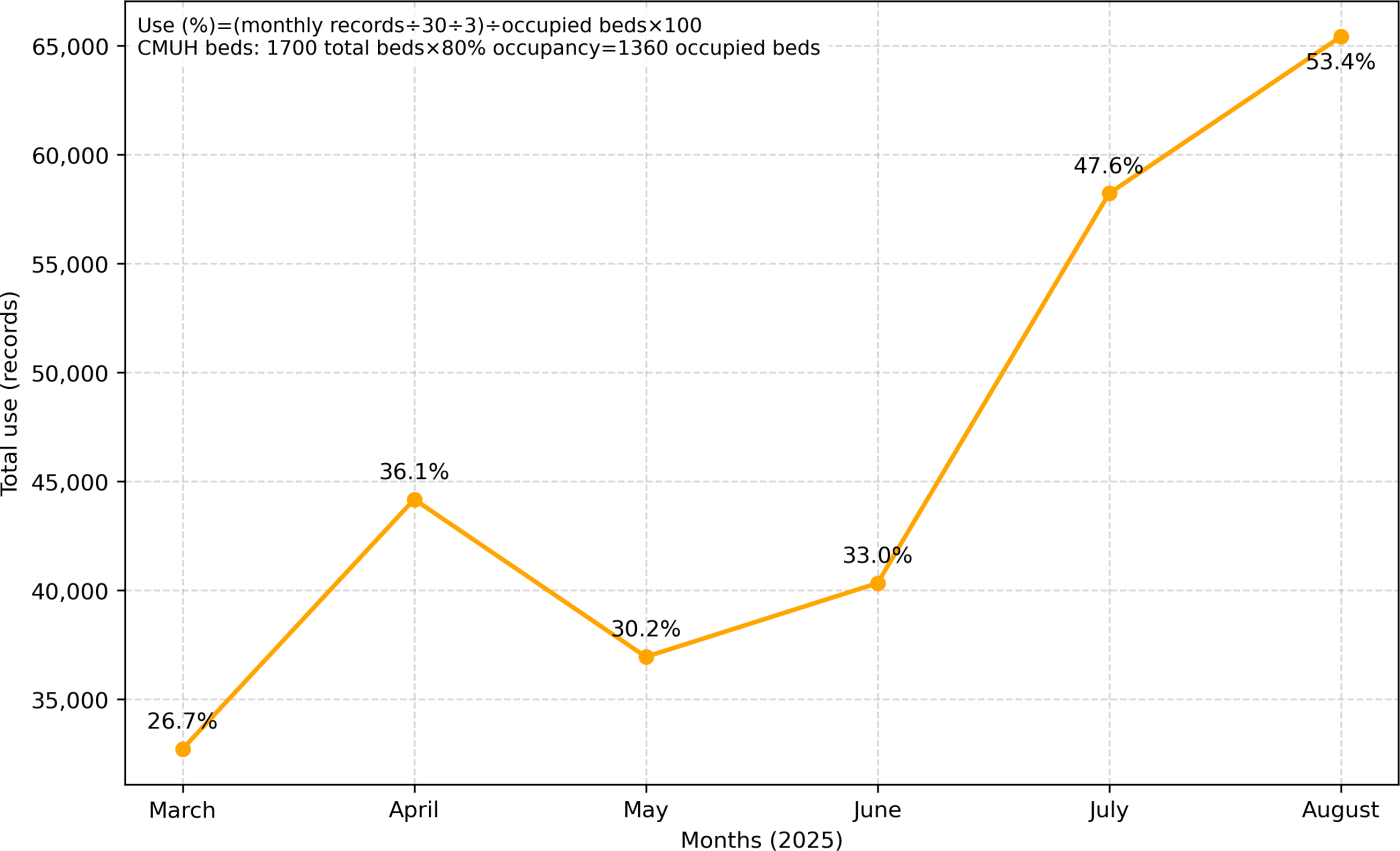
Monthly system use at China Medical University Hospital (CMUH) from March 2025 to August 2025.

Following deployment, the system was increasingly incorporated into routine documentation at CMUH. Monthly use nearly doubled between March 2025 and August 2025, rising from 32,724 to 65,417 valid DART notes generated and uploaded to the HIS through voluntary use of the system (Figure 3). This upward steady increase indicates expanded use across clinical units during the initial deployment period.

User feedback was collected from a subset of nursing staff who voluntarily provided evaluations (n=120, representing approximately 12.2% of the 982 nurses actively working in the 44 participating units across wards and intensive care departments). Among these participants, 91 (75.8%) nurses reported a favorable experience, whereas 29 (24.2%) expressed dissatisfaction. Positive feedback emphasized reductions in manual transcription burden and improvements in documentation efficiency, whereas negative feedback primarily concerned occasional recognition inaccuracies and the continued need for verification. Although the feedback sample does not represent all users, it reflects the perspectives of frontline staff actively engaged with the system. Detailed departmental participation is provided in Multimedia Appendix 2. Collectively, these findings demonstrate that the system is both technically feasible and operationally acceptable in real-world clinical environments.

## Discussion

This study designed and implemented a speech-based documentation system that integrates a domain-adapted ASR model with an LLM to generate structured nursing records and evaluated its performance in real clinical settings. The fine-tuned Whisper large-v2 model achieved a MER of 6.6%, demonstrating high accuracy for Mandarin-English nursing speech. With schema-guided LLM structuring, the system reached an *F*_1_-score of 0.82, which was statistically noninferior to human-transcribed input, and exhibited a low hallucination frequency (2.51%). These findings indicate that domain-adapted ASR combined with LLM-based structuring can produce structurally consistent draft documentation that, alongside routine human verification, reduces cognitive and transcription burden in routine practice.

Field deployment demonstrated that the system could be incorporated into routine clinical workflows. By enabling point-of-care mobile dictation directly at the bedside, the system effectively replaced the traditional, error-prone workflow of delayed, memory-reliant documentation at the nursing station, directly addressing the critical pain points exacerbated by high nurse-to-patient ratios. System use doubled over the 6-month observation period. Because the hospital operates at a consistently high and stable capacity, this increase in absolute volume was not driven by fluctuations in patient census. Rather, as the system was deployed as an optional tool, this steady growth reflects successful voluntary adoption, supported by continuous iterative optimizations based on user feedback. Feedback from nursing staff further supported its usability. Most respondents reported reduced manual effort and improved efficiency, while negative feedback focused on recognition accuracy and the need for verification, which aligns with the human-in-the-loop design. Participation across 6 major ward categories indicates that the system was used in a wide range of clinical settings.

Several limitations warrant consideration. First, the dataset was modest and sourced from a single institution, which may constrain generalizability. Second, user feedback was voluntary and may overrepresent individuals who were more engaged with the system, and long-term patterns of use were not assessed. Third, our evaluation of structural content accuracy and hallucination relied primarily on an LLM-as-a-judge framework (RAGAS). The primary purpose of this evaluation was to conduct a strict semantic fidelity check, ensuring that the LLM strictly formatted the ASR transcripts into the DART schema without altering, inferring, or omitting clinical facts. Although RAGAS performs robust semantic contradiction checks, traditional non-LLM baselines (eg, exact string matching) were deemed unsuitable because of the heavy code-switching and spoken-to-written paraphrasing inherent in nursing documentation. Although senior nursing staff qualitatively reviewed the generated drafts within our human-in-the-loop workflow, the lack of a formal, quantitative manual fidelity evaluation on a stratified sample of high-risk note types by nursing professionals remains a limitation. Future work should include multisite validation; evaluation of interoperability with diverse HIS environments; dedicated quantitative manual safety reviews by senior nurses to further validate that the LLM does not alter critical nursing parameters (eg, medication dosages and invasive device settings); and longitudinal assessment of impacts on documentation quality, workflow efficiency, and staff satisfaction.

## Supplementary material

10.2196/88567 Multimedia Appendix 1 Prompt templates for DART (data, action, response, and teaching) versions 1 and 2 used to guide large language model–based structuring of nursing narratives into DART records.

10.2196/88567 Multimedia Appendix 2 Distribution of user feedback by ward category.

## Abbreviations

ASR: automatic speech recognition
CMUH: China Medical University Hospital
DART: data, action, response, and teaching
HIS: hospital information system
LLM: large language model
MER: mixed error rate
RAGAS: Retrieval-Augmented Generation Assessment
