# Supplementary material for: Voice-Based Structured Nursing Documentation Using Automatic Speech Recognition and Large Language Models: Development and Evaluation Study
Source: JMIR Nurs. 2026 Jun 5;9:e88567. doi: 10.2196/88567 (PMC13240795; doi:10.2196/88567)
Supplement: Multimedia Appendix 2 [file nursing-v9-e88567-s002.docx]

Multimedia Appendix 2

Table S1. Distribution of user feedback by ward category.

| Catagory | Like (n^a^) | Dislike (n) | Like Ratio (%) |
| --- | --- | --- | --- |
| B^b^ (Pediatric Wards) | 28 | 5 | 84.8 |
| H^c^ (Surgical Wards) | 22 | 7 | 75.9 |
| C^d^ (Medical Wards) | 24 | 5 | 82.8 |
| E^e^ | 3 | 1 | 75.0 |
| A/I^h^ | 4 | 1 | 80.0 |
| Intensive Care Units^i^ (ICU / CCU / SCU / NSCU / RCU / RCC / MICU / RICU) | 39 | 10 | 79.6 |

^a^n: number of user feedback entries. Like ratio (%) was calculated as Like ÷ (Like + Dislike) × 100.

^b^B (Pediatric Wards) – Children’s Hospital pediatric units, including 6B, 7B, 8B, BR, NICU, PICU, and SBR.

^c^H (Surgical Wards) – Adult surgical wards, including 6H, 7H, 8H, 9H, 10H, 11H, 12H, 13H, and 14H.

^d^C (Medical Wards) – Adult medical wards, including 8C, 10C, 11C, 12C, 13C, 14C, 15C, 16C, 17C, 18C, and 20C.

^e^E – Emergency wards (9E and 10E).

^h^A/I – General and isolation wards (5A and 5I).

^i^Intensive Care Units – Intensive and step‐down units, including MICU, RICU, CCU, burn center, neurosurgical ICU (NSCU), respiratory care centers (RCC1–3), respiratory care unit (RCU2), surgical ICUs (SCU1–2), and the stroke and neurocritical care ICU.
